# Prevalence of brucellosis in livestock of African and Asian continents: A systematic review and meta-analysis

**DOI:** 10.3389/fvets.2022.923657

**Published:** 2022-09-09

**Authors:** Kuralayanapalya P. Suresh, Sharanagouda S. Patil, Akshata Nayak, Himani Dhanze, Shinduja Rajamani, Chandan Shivamallu, Charley A. Cull, Raghavendra G. Amachawadi

**Affiliations:** ^1^Spatial Epidemiology Laboratory, Indian Council of Agricultural Research (ICAR) - National Institute of Veterinary Epidemiology and Disease Informatics (NIVEDI), Bengaluru, Karnataka, India; ^2^Virology Laboratory, Indian Council of Agricultural Research (ICAR) - National Institute of Veterinary Epidemiology and Disease Informatics (NIVEDI), Bengaluru, Karnataka, India; ^3^ICAR-Indian Veterinary Research Institute, Bareilly, India; ^4^Division of Biotechnology and Bioinformatics, School of Life Sciences, JSS Academy of Higher Education & Research, Mysuru, Karnataka, India; ^5^Midwest Veterinary Services, Inc., Oakland, NE, United States; ^6^Department of Clinical Sciences, College of Veterinary Medicine, Kansas State University, Manhattan, KS, United States

**Keywords:** brucellosis, meta-analysis, meta-regression, prevalence, systematic review, zoonosis

## Abstract

Brucellosis is a highly contagious bacterial disease that mainly affects ruminants, but it may affect equines, canines, and felines. The disease is of utmost significance from an economic standpoint in countries where there is no national brucellosis prevention and eradication policy in operation. A systematic review was done to estimate disease burden, incidences, prevalence, and geographical distribution critical in planning appropriate intervention strategies for the control and prevention of Brucellosis. Research articles that were published during the period 2000–2020 were considered for this study after reinforced scrutiny by two independent authors. Meta-regression was used to examine heterogeneity, and subgroup and sensitivity analyses were used to calculate residual heterogeneity and the pooled prevalence of Brucellosis in livestock. Confounders such as geography, a diagnostic test, and species had the greatest *R*^2^ values of 17.8, 8.8, and 2.3%, respectively, indicating the presence of heterogeneity and necessitating more research into sensitivity and subgroup analysis. The combined pooled prevalence of brucellosis in both Asia and African countries was 8% when compared to 12% in the Indian livestock population. The findings of our systematic review and meta-analysis indicate that brucellosis continues to be an important animal and public health concern in developing countries of Asia and Africa, as evidenced by the prevalence rate of brucellosis in these regions. Our findings suggested that well-planned epidemiological surveillance studies in different geographic settings are needed to generate reliable data on disease burden including the economic loss in Asian and African countries.

## Introduction

Brucellosis is an endemic zoonotic disease in Asian and African countries and has a significant impact on both animal and human health. It still remains as one of the major public health concerns throughout developing countries, accounting for an annual occurrence of over 500,000 cases (1). In most developed countries, the infection has been contained and eliminated, however, it remains endemic in Africa, Latin America, and Asian continents (2). Infected animals exhibit clinical signs that are of economic significance to stakeholders and include reduced fertility, abortion, poor weight gain, lost draft power, and a substantial decline in milk production. In humans, brucellosis typically manifests as a variety of non-specific clinical signs. Chronicity and recurring febrile conditions, as well as devastating complications in pregnant women are common sequelae. In some European and North American countries, the disease has been eradicated due to restrictions posed on the international trade of animals and animal products. Compared to Europe, North America and other developed areas of the world, the main burden of the disease is felt in the Mediterranean, South and Central America, Africa, Asia, the Indian subcontinent, Eastern Europe and the Middle East, especially Syria, Iraq, Egypt, Turkey and Iran. In India, bovine brucellosis is prevalent and appears to be on the rise lately, possibly due to increased trade and rapid livestock movement (3).

The disease is caused by Gram-negative facultative intracellular bacterial organisms of the genus *Brucella*. Among them, three bacterial species that are highly pathogenic are *Brucella abortus, Brucella melitensis*, and *Brucella suis* (3). Likewise, the other virulent species of Brucella are *Brucella canis, Brucella ovis, Brucella neotomae, Brucella microti*, and two species namely *Brucella cetaceans* and *Brucella pinnipedialis* are of marine origin (4). Among these, *B. abortus* is highly pathogenic, can survive intracellularly, and can infect ruminants for a long time period. Some infected animals remain asymptomatic, with no clinical symptoms of latent infection, thereby sustaining the disease in a population (4).

Bovine brucellosis causes huge loss to the dairy industry; however, there is a dearth of comprehensive economic studies. Loss of 6%−10% of the income per animal was reported in Africa (5). Samartino (6) estimated annual economic losses of around US$ 60 million in Argentina. In Nigeria, losses were estimated at US$ 575,605 per year. Losses resulting from Brucella infection are even lesser documented in tropical Asia. As per the National Animal Disease Referral Expert System (NADRES) there are 461 deaths due to brucellosis are reported from 2000 to 2021.

The overall burden of disease is assessed using the disability-adjusted life year (DALY), a time-based measure that combines years of life lost due to premature mortality (YLLs) and years of life lost due to time lived in states of less than full health, or years of healthy life lost due to disability (YLDs). Economic losses of human brucellosis were calculated based on the official records and the data from epidemiological surveys. These data were used to estimate the disability-adjusted life years (DALYs) due to human brucellosis. The annual median losses due to human brucellosis were estimated to be Rs 627.5 million [uncertainty interval (95% UI) Rs 534.8–741.2 million; US $ 10.46 million] with a loss of Rs 442.3 million (95% UI 371.0–516.0; US $ 7.37 million) among adults and Rs 185.0 million (95% UI 124.0–255.0; US $ 3.08 million) among children. Human brucellosis in India is estimated to cause a loss of 177 601 (95% UI 152 695–214 764) DALYs at the rate of 0.15 (95% UI 0.13–0.17) DALYs per thousand persons per year. The DALYs were found to be 0.29 (95% UI 0.08–0.70) per thousand persons per year in the occupational and 0.13 (95% UI 0.06–0.18) in the non-occupational adult population (7).

Infection in ruminants usually occurs after consumption of contaminated milk, feed, water, or grazing forage, direct contact with infected animals, uterine secretions or aborted fetuses, and vertical and sexual transmission (8, 9). Cattle and buffalo, the major reservoirs, are specifically infected by *B. abortus* although it is possible that other domestic and wild animals may acquire the infection (10). Abortion, miscarriage, stillbirth, the birth of weak calves, and the presence of epididymitis and orchitis in adults are the key clinical indications (11).

The introduction of animals from contaminated herds into uninfected herds may lead to infection of healthy animals (12). After their first abortion, infected animals will stay as contagious carriers and continue to transmit the disease (13). The methods to control and eliminate brucellosis are based on vaccination, controlling movements, and testing and isolating serologically positive animals. Four vaccines that have been approved against brucellosis for use in animals by the OIE are (i) *B. abortus* strain 19 (S19), (ii) *B. abortus* strain RB51 (RB51) (iii) *B. melitensis* strain Rev. 1 (Rev. 1), and (iv) *B. suis* strain (S2). Serology remains an important tool employed for the diagnosis of brucellosis. In cattle, Rose Bengal Plate Test (RBPT) is used as a screen test followed by testing positive sera with complement fixation test or ELISA for confirmation. The milk ring test (MRT) could be used for identifying infected dairy herds with good results followed by sero-testing individual animals.

The MRT is generally used for primary screening and it is mandatory to use the other tests for confirmatory diagnosis (14–16). Morgan stated that the MRT can be used in combination with the existing tests and not alone (17, 18). In general, the MRT has been shown in other studies to have high sensitivity but lower specificity, hence comparatively less used for detection (18). Bacterial and antibody detection played vital roles in the brucellosis eradication program since its first report in 1934, and there have been various improvements in the field of disease diagnostics in the earlier decades (18).

To set priorities for livestock health policy, such as funding for veterinary health interventions and planning for curbing the burden of brucellosis, it is necessary to have accurate data on the prevalence of brucellosis in livestock in all countries. With an increase in the submission of research articles, the value of systematic review and meta-analysis for summarizing the results is greatly acknowledged. Meta-analysis is a statistical method that combines and synthesizes multiple studies and integrates their results (19). It also considers the sample size of various studies and provides a precise estimate of prevalence (20). Data synthesized from the meta-analysis are usually more beneficial than the results of narrative reviews, decisions are transparent and statistical analysis yields an objective measure of the integrated quantitative evidence (20). The systematic review uses systematic methods to identify, select and analyze the primary studies both qualitatively and quantitatively, while meta-analysis is part of a systematic review and employs statistical methods to integrate the results from multiple primary research studies. The main objectives of our study were to (i) estimate the more valid, generalizable summary estimates of the prevalence of brucellosis, (ii) identify and provide information on factors or covariates that affect the prevalence, and (iii) identify the areas for further research. Meta-analysis represents a powerful way to summarize and effectively provides a more valid pooled estimate. When the incidence of the disease is high and there is no specific treatment available, the role of surveillance programs becomes imperative to enforce effective prevention and control strategies and helps policy makers for policy changes and practices and further research. Result of this study could be input for the researcher and policy maker about the brucellosis disease burden, thereby supporting the process of identification of priorities in veterinary and public health care, prevention or intervention strategies, and control policies.

## Materials and methods

### Literature search strategy

One of the key distinctions between conventional narrative analysis and a systematic review is a structured search of the literature. The goal of a systematic review is to find as many important studies on the subject as possible. Prior to performing a literature search, a systematic search plan was developed and recorded in the research procedure to accomplish the same. A systematic search for published articles reporting prevalence data for bovine brucellosis in cattle, buffaloes, sheep, and goats worldwide and in India was conducted for our study. The databases like PubMed (Link), Google Scholar (Link), Science Direct, Scopus, and Consortium for e-Resources in Agriculture (CeRA, India) were used to comprehensively capture articles published both nationally and internationally. After examining common Medical Subject Headings (MeSH) terms for pre-identified and relevant publications, the following search terms were used across all four databases: “Brucellosis” OR “Bovine Brucellosis”) AND (cows OR cattle OR bovine OR sheep OR goat) AND (epidemiology^*^ OR prevalence^*^ OR incident^*^ OR surve^*^) AND (world OR Africa OR Asia OR India). The articles were restricted to the English language only and were published between 2000 and 2020 (for a period of 20 years). Additional articles were also identified manually from the reference lists of downloaded articles by “back-reference search.”

### Exclusion and inclusion criteria

The formulation of inclusion and exclusion standards to assess whether or not literature articles are eligible for systematic review and meta-analysis should concentrate on two issues: the importance of research questions and the consistency of methodology. The most critical element in determining inclusion criteria is the importance of empirical issues, while analytical consistency determines the exclusion criteria (21). Included studies reported the prevalence of Brucellosis in cattle, buffalo, sheep, and goats in worldwide countries based on commonly accepted methods for the diagnosis of Brucellosis. Prevalence studies that examined the effects of Brucellosis control strategies were excluded in order to avoid the introduction of potential sampling bias, as the primary aims of these studies were to compare the effectiveness of control strategies. Primary screening of articles was done by reading the abstract alone, then the methodology, and finally the results section of the individual article was studied and screened. Our study period was restricted to articles and reports published from 2000 to 2020. Those articles published in the English language only were included for the final systematic review and meta-analysis. Both, Zotero 5.0 and Rayyan QCRI (the Systematic Reviews web app) were used for systematic review. Excluded studies were reviews, case studies, massive sample sizes, non-confirmatory diagnostic tests, and studies not mentioning the species of the animal testing. Finally, all included studies were cross-sectional and diagnostic test accuracy (DTA) studies. The PRISMA guidelines were followed to extract the relevant articles (22).

### Data extraction and management

A systematic review and meta-analysis are increasingly common (21). Hence, data collection is a vital part of systematic review and meta-analysis, and it also bridges the gap between review and meta-analysis (21, 23). This ensures that data cleaning and analysis are as easy, reasonable, and accurate as possible. Lack of coordination between authors and data analysts can lead to errors and incorrect results or inferences in systematic reviewing (23). In the present study, two authors independently extracted the data on bibliographic, demographic, and study outcomes in pre-test format, and any disagreements between the authors were solved by discussion. Two authors independently reviewed all publications before comparing their respective data forms. The data recorded were study characteristics including author, publication year, study period, location of study, the diagnostic test used, and criteria for positivity, sample size, the prevalence of brucellosis in cattle, buffalo, sheep, and goats. For the formal review of all articles generated, an initial screening for inclusion was made based on the titles, abstracts, and publications. Those specifying different species, countries, or other diseases were excluded. Additionally, full texts were read for any prevalence data that could be extracted. Finally, data were extracted from 80 eligible articles, listed in the Supplementary File S6.

### Quality of study bias assessment

Inter-rater agreement is an important consideration for researchers when developing scales used to measure the quality assessment of any construct. Aiken's V was proposed to summarize the agreement ratings from a panel of expert judges (24). The studies were assessed for bias by using Inter-rater agreement between two authors on 8 item-structured questionnaires using the modified risk of bias tool (25, 26). The agreement index proposed by Aiken is formulated as follows: (1) where V is the item validity index; s is the scores assigned by each rater minus the lowest score in the used category *s* = *r* – lo, with *r* = rater category selection score and lo the lowest scores in the scoring category; *n* is the number of raters, and c is the maximum score in the grading scale, *S* is the sum of *s* for the *n* raters (27). Hence, the Aiken V index is given by the formula:


V = S/([n∗(c−1)])

where V ranges from 0 to 1.0. A score of 1.0 is interpreted by all raters, giving the item the highest possible rating. The reviewers were blinded with respect to study authors, institutions, or journals. The ratings of the questionnaire were in Likert scaling format with a 5-point scale where minimum score (1) represents very unlikely and maximum score (5) represents very likely (27). Kappa Index was used to find the agreement of two independent authors in regard inter-rater reliability of scale.

The two authors independently rated the collected studies based on questionnaires with Likert's scaling of 1–5. The average score of all the studies was taken as the final score for an individual article. The Inter-rater reliability of the scale was established using Kappa statistics. The rating scores obtained by the two independent authors were subjected to the calculation of Aiken's V Index for agreement. The study quality is confirmative and acceptable if the Aiken V Index was more than 0.7 and above.

### Data analysis

#### Systemic review and meta-analysis

A sound understanding of the methodology is essential for the effective conduct of systematic review and meta-analysis. In order to reduce bias and ensure transparency in compiling and evaluating the information found in the published literature, systematic review procedures have been developed (28). To perform a competent systematic review and meta-analysis, many recommendations and checklists are available, such as the Preferred Reporting Items of Systematic Reviews and Meta-Analysis (PRISMA) (29).

Meta-analysis is a statistical research process used to assimilate various studies to calculate an overall summary estimate. Cochran's *Q* statistic and Higgin's I statistics were calculated to test heterogeneity among the included studies (28, 30). Predominant in the meta-analysis, Fixed Effects model and the Random-effects model are widely used (25). When a large number of studies are included and there are few variations among them, *I*^2^ will be low, hence the Fixed Effects Model may be used (31). When a large number of studies include significant heterogeneity, the Random Effects Model can be used to explain the distribution among the studies (31). All quantitative analyses were performed in R Open-source scripting software (version 4.0.2, R Foundation for Statistical Computing, Vienna, Austria). The R packages used for the analysis were “meta” “metar,” “metafor,” “qdap,” “dmetar,” “mass” and “openxlsx” packages^1^. The variability among studies relates to heterogeneity. Heterogeneity may arise from random chance in analytical methods, such as disparities in research design & screening, technique, criterion for inclusion and exclusion, variations in prevalence rates, etc. (32). Where the heterogeneity between studies is visible and huge, the use of meta-analytic study pooling is no longer important and hence not recommended (32). The degree of heterogeneity in a meta-analysis mostly decides the effort in reaching general interpretations. This degree might be estimated by assessing the variance between the different studies (33). A test for the existence of heterogeneity subsists but depends on the number of studies in the meta-analysis (32). Indices H and *I*^2^, are usually calculated to summarize the impact of heterogeneity among included studies (32). Inconsistency (*I*^2^), a measure of the degree of inconsistency (ranging from 0–100%), and heterogeneity are assessed using Cochran's *Q* test as well as Higgins' *I*^2^. The importance of the observed values of *I*^2^ depends on (i) the magnitude and direction of estimates and (ii) the strength of evidence for heterogeneity across studies in a meta-analysis. *I*^2^ is preferable to test for heterogeneity in judging the consistency of evidence. If *I*^2^ < 50 it signifies least heterogeneity, *I*^2^ > 50% characterizes least-moderate heterogeneity, and *I*^2^ > 95% indicates high heterogeneity (30, 31). It is important to consider the consistency of the results of different studies. If confidence intervals for the results of individual studies (generally depicted graphically using horizontal lines) have poor overlap, this generally indicates the presence of statistical heterogeneity (27). More formally, a statistical test for heterogeneity is available. This chi-squared (χ^2^, or Chi^2^) test is included in the forest plots in Cochrane reviews (32, 33). It assesses whether observed differences in results are compatible with chance alone. A low *p*-value (or a large chi-squared statistic relative to its degree of freedom) provides evidence of heterogeneity (variation in effect estimates beyond chance) (30)^2^. In the present study, the *p*-value of <0.05 is considered to have the presence of heterogeneity.

#### Meta-regression

Meta-regression is conducted to analyze the characteristics of included studies that might influence the estimates, mainly when large studies have been included for analysis. Large-scale investigations have more impact as they are weighted by the exactness of their individual impact estimate (34). It is quick to consider the leftover heterogeneity among intercession impacts of those which are not exhibited by the variable (34). The relation between the outcome variable and the explanatory variable was defined by the regression coefficient obtained (the potential effect modifier) (34). For categorical explanatory variables that can be further used for subgroup comparisons, meta-regression can also be used to analyze variations (35). The *p*-value estimates the statistical significance of each regression coefficient. Meta-regression reduces the number of tests and estimations as compared to subgroup analysis; hence the power of analysis is greater, and the probability of false-positives findings is reduced (35). In the present study, the factors like region, species, a test of diagnosis, sample size, and quality of bias score were studied by meta-regression for quantifying the amount and degree of heterogeneity.

#### Subgroup analysis

Subgroup analysis was performed for the covariates like region, diagnostic test, and different species of livestock due to their significant contribution to the heterogeneity. In our study, the subgroups were stratified based on the articles from different geographic locations (continent-wise, species-wise, and test/method used to confirm the Brucellosis was considered). We tried to generate pooled estimates by employing subgroup and sensitivity analysis with respect to reduce *I*^2^, a heterogeneity compounded in the study.

#### Statistical modeling

The modeling through meta-analysis of 80 studies indicated the significant presence of heterogeneity leading to further analysis by meta-regression for identifying the statistically significant contributing factors.

Based on the outcome of meta-regression, *R*^2^ values obtained gives an insight into which variables require subgroup analysis. Subgroup analyses involve splitting all the data into relevant subgroups in order to compare the different studies (subsets may be done for studies from different geographical locations, tests/methods used to confirm the disease, different species of animals, year of publication, countries, etc.).

Sensitivity analysis is very important and useful as it improves the robustness of estimates and prediction by studying the model response to changes in input variables. Sensitivity analysis aims at improving knowledge and this analysis reduces the uncertainties of the parameters of the assessment, and then decisions about the phenomenon under study can be ascertained. In the present study, sensitivity analysis was performed to identify the studies which contribute to overall heterogeneity and measure the robustness of meta-analysis findings.

### Publication bias

The extent of publication bias in the selected studies was measured and demonstrated by a funnel plot. The funnel plot that was generated showed the heterogeneity among the included studies (36). Additionally, we conducted Egger's test to assess the level of publication bias (37).

## Results

### Search results

The literature search identified 855 articles from five electronic databases. The databases like PubMed (268 articles), Google Scholar (392 articles), Science Direct (52 articles), Scopus (95 articles), and Consortium for e-Resources in Agriculture (CeRA, India; 48 articles) were used to comprehensively capture articles published both nationally and internationally.

**Figure 1:**
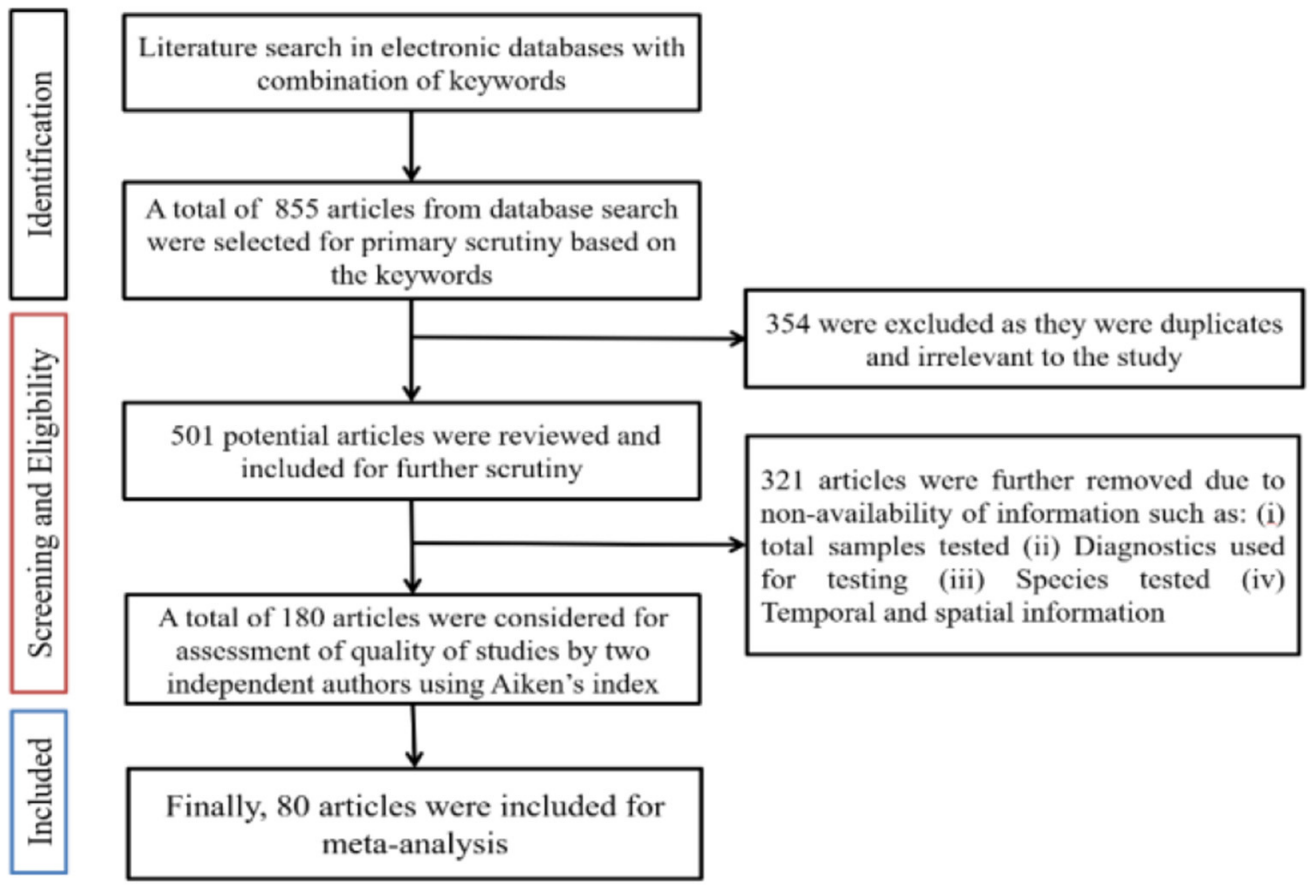
Flowchart showing study inclusion and exclusion criteria.

After removing the duplicates and irrelevant articles, 501 was retained for further analysis. A comprehensive evaluation of titles and abstracts resulted in the exclusion of 321 articles. A full article review was independently conducted by two authors on the remaining 180 articles for assessment of the quality of studies using Aiken's Index for an agreement finally, we have considered a total of 80 articles, wherein 28 articles were from seven countries of the African continent and 52 articles from nine countries of the Asian continent, of which 29 articles were from India and included for conducting meta-analysis. The studies included in this review are provided in the Preferred Reporting Items for Systematic Reviews and Meta-analysis (PRISMA) flowchart (Figure 1). Data extraction and the inclusion/exclusion criteria were in accordance with the PRISMA checklist that is recorded in Supplementary File S1.

### Assessment of quality of studies

Two authors independently rated the 180 studies based on questionnaires with Likert's scaling of 1 to 5 (score 1 being less relevant and 5 being the more relevant). The average score of all the studies was taken as the final score for an individual article. The Inter-rater reliability of the scale was established using the Kappa statistic. The rating scores obtained by the two independent authors were subjected to the calculation of Aiken's V Index for agreement. The study quality is confirmative and acceptable if the Aiken V Index was more than 0.7 & above, finally, 80 studies were retained for the conduct of meta-analysis (Table 1 and Supplementary File S6). The average score obtained on 8 items of the scale for 80 articles scored by two independents authors were analyzed using Wilcoxon Signed rank test, which was found to be non-significant (*p* > 0.05) indicating the agreement of the two authors, and hence the 80 articles were included for the conduct of meta-analysis.

**Table 1:** Interrater agreement testing between two raters in using the risk of bias tool.

**Sl. no**.	**Validation procedures**	Author 1^*^	Author 2^*^	**KAPPA (95%CI)**
**External validation**				
1	Was the study's target population representative of the national population with respect to relevant variables?	4.41	4.46	0.601 (0.44: 0.74)
2	How were the samples selected, randomly or was census undertaken?	4.39	4.34	0.821 (0.69:0.94)
3	Was the probability of bias minimal?	4.44	4.38	0.700 (0.52:0.87)
**Internal validation**				
4	Was the data collected directly from the subjects?	4.19	4.30	0.786 (0.64:0.92)
5	Was an acceptable case definition used in the study?	4.44	4.49	0.791 (0.66:0.92)
6	Was the used study method to measure parameter valid and reliable?	4.38	4.34	0.840 (0.73:0.96)
7	Was the same mode of data collection used?	4.49	4.33	0.805 (0.67:0.93)
8	Summary on the overall risk of study bias	4.15	4.39	0.762 (0.58:0.94)

* Average score of two independent authors and Kappa Index (95%CI) score of 80 articles included for meta-analysis.

### Publication bias

**Figure 2:**
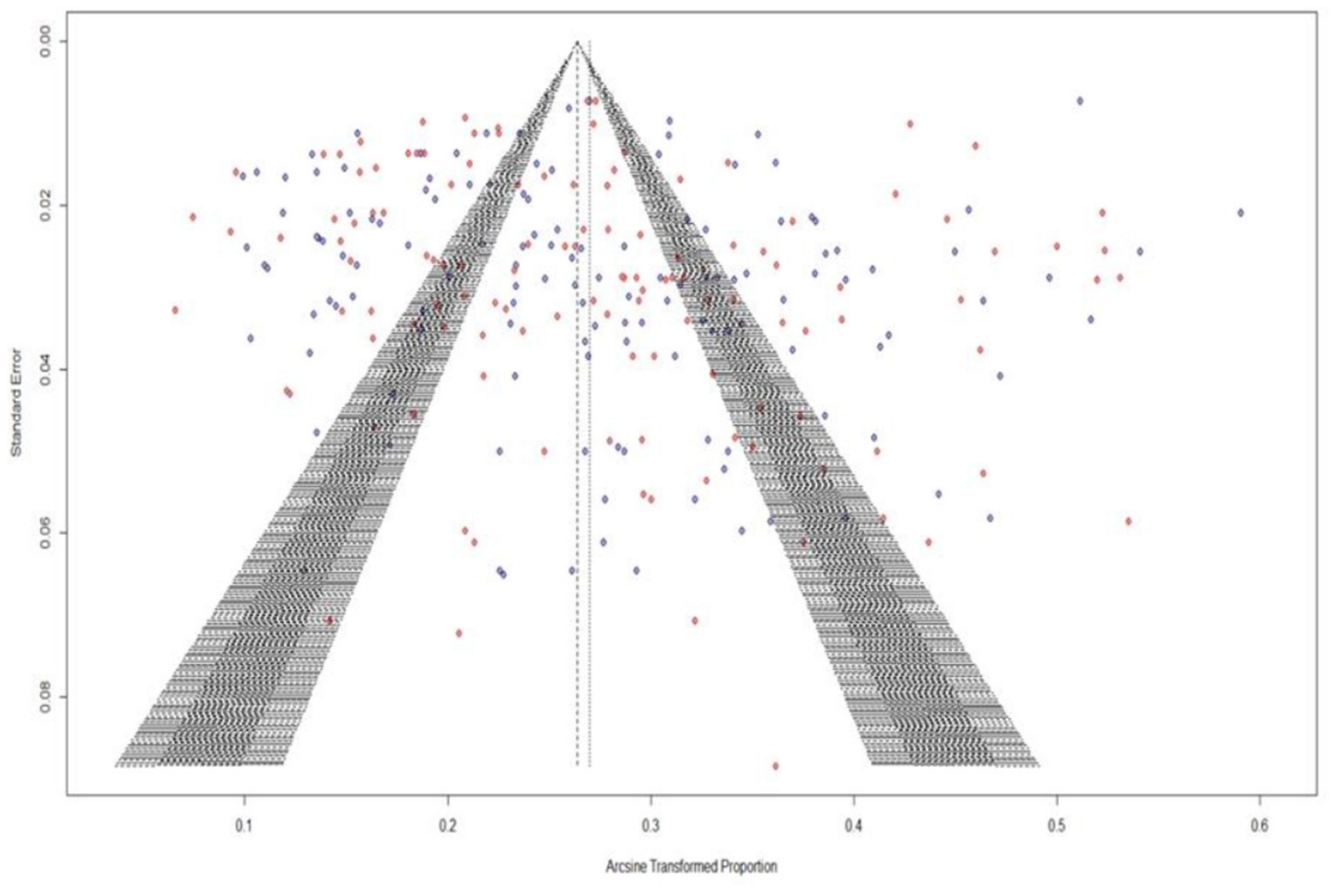
Publication bias among studies is shown in funnel plots showing asymmetry and heterogeneity.

The Figure 2 represents the Funnel plot for ascertaining the presence of publication bias. The careful visualization of funnel plot indicated the presence of minor publication bias. Further, funnel plot for asymmetry was assessed with significance testing by rank correlation test by Kendall's Tau (Kendall's tau = 0.2090, *p* < 0.0001) and Egger's test (*z* = 12.32, *p* < 0.010) indicating the presence of asymmetry due to publication bias which may be attributed to the specific type of heterogeneity leading to smaller studies showing effects that differ significantly from larger studies. To deal with the presence of publication bias, we have employed the meta-regression with sample size as the risk of bias factor, proving the non-significance (*p* > 0.05) nullifying the effect of publication bias in the study.

### Meta-regression to identify the factors affecting the heterogeneity

Meta-regression revealed strong evidence of high heterogeneity among selected studies on the prevalence of brucellosis in livestock. Univariate meta-regression was conducted to identify potential covariates that likely affect the magnitude and direction of the overall estimate. However, meta-regression with the region [regression coefficient (Qm) = 79.83, *p* < 0.001], detection techniques (tests; Qm = 59.91, *p* < 0.001) and species (Qm = 11.65, *p* = 0.010)] suggested that the covariates had significant effect on the heterogeneity between studies (Table 2). *R*^2^ was found to be highest among variables such as region (17.76%), the test used in diagnosis (8.78%), and species (2.26%). Finally, it was concluded that the subgroup and sensitivity analysis is required for region-wise, diagnostic tests-wise and species-wise variables for further fine-tuning of prevalence rates of brucellosis.

**Table 2:** Table showing the Unitarians meta-regression analysis of Brucellosis in livestock.

**Predictors**	**Estimate**	**SE**	***z*-Value**	**τ^2^**	***I*^2^ (%)**	***H*^2^ (%)**	***R*^2^ (%)**	**Qm**	***p*-Value**
Region (Ref)	0.295	0.013	22.878	0.019	97.66	42.72	17.76	79.83	<0.0001
Test	0.328	0.150	2.190	0.021	97.81	45.61	8.78	59.91	<0.0001
Species	0.300	0.017	17.766	0.023	98.02	50.49	2.26	11.65	0.01
Quality	0.422	0.072	17.766	0.023	98.06	51.46	0.36	2.25	0.07
Sample Size	0.324	0.017	33.899	0.153	98.04	51.01	0.49	3.22	0.07
Year	2.918	3.170	0.920	0.024	98.07	51.70	0.00	0.67	0.41

### Subgroup analysis

During the investigations of the results of subgroup analysis, it was observed that few studies exhibited outliers.

### Prevalence of brucellosis in Asia and Africa

Sub-group analysis revealed that the African continent showed a prevalence of 8% (95% CI: 7–10%, *I*^2^ = 96%, τ^2^ = 0.0104, *p* < 0.001) from the 58,509 tested samples (Table 3a). In Asia, the pooled prevalence of Brucellosis was also found to be 8% (95% CI: 7–9%, *I*^2^ = 96%, τ^2^ = 0.0149, *p* < 0.001) from a sample size of 142,638 animals (Supplementary Figure S2). Brucellosis is an endemic zoonotic disease in most of the developing world that causes devastating losses to the livestock industry and small-scale livestock holders.

**Table 3:** Prevalence of Brucellosis stratified according to (a) Continent-wise (b) Diagnostic test-wise (c) Species-wise for sub group analysis.

**(a) Continent-wise stratification**				
**Names of continent**	**Prevalence % (95% CI)**	***I*^2^ (%)**	**τ^2^**	**Model**
Africa	8.0 (7.0–9.0)	96	0.0104	REM
Asia	8.0 (7.0–9.0)	96	0.0149	REM
**(b) Diagnostic test-wise stratification**				
**Names of the test**	**Prevalence % (95% CI)**	**I**^**2**^ **(%)**	**τ^2^**	**Model**
ELISA	7.0(6.0–8.0)	97	0.0122	REM
PCR	11.0(2.0–26.0)	79	0.0317	REM
RBPT	8.0(7.0–9.0)	93	0.0085	REM
MRT	7.0(4.0–11.0)	94	0.0107	REM
Agglutination Tests	7.0(6.0–8.0)	94	0.0115	REM
CFT	10.0(8.0–11.0)	75	0.0009	REM
LFA & FPA	4.0(3.0–6.0)	50	0.0019	FEM
Riv. Test	4.0(3.0–5.0)	56	0.0005	FEM
**(c) Species-wise stratification**				
**Names of Species'**	**Prevalence % (95% CI)**	***I**^2^* **(%)**	**τ^2^**	**Model**
Buffalo	6.0 (5.0–8.0)	90	0.0085	REM
Cattle	8.0(7.0–9.0)	97	0.0124	REM
Goat	6.0(5.0–7.0)	82	0.0054	REM
Sheep	7.0(6.0–8.0)	90	0.0038	REM

REM, random effects model; FEM, fixed effect model.

### Diagnostic test-wise and species-wise analysis

Stratification based on confirmatory test revealed that samples tested by PCR and CFT showed the highest prevalence of 11% (95% CI: 2%−26%) and 10% (95% CI: 8%−11%), respectively (Table 3b). Detection by ELISA confirmed a prevalence of 7% (95% CI: 6%−8%) whereas, the samples tested by RBPT, MRT, Agglutination test revealed the prevalence of 8, 7, and 7%, respectively (Supplementary Figure S3).

Species-wise stratification showed that cattle had the highest prevalence of 8% (95% CI: 7%−9%, *I*^2^= 97, τ^2^ = 0.0124, *p* = 0, Table 3c) followed by sheep with 7%, both buffalo and goat had prevalence of 6% each (Supplementary Figure S4).

### Prevalence of brucellosis in India

**Figure 3:**
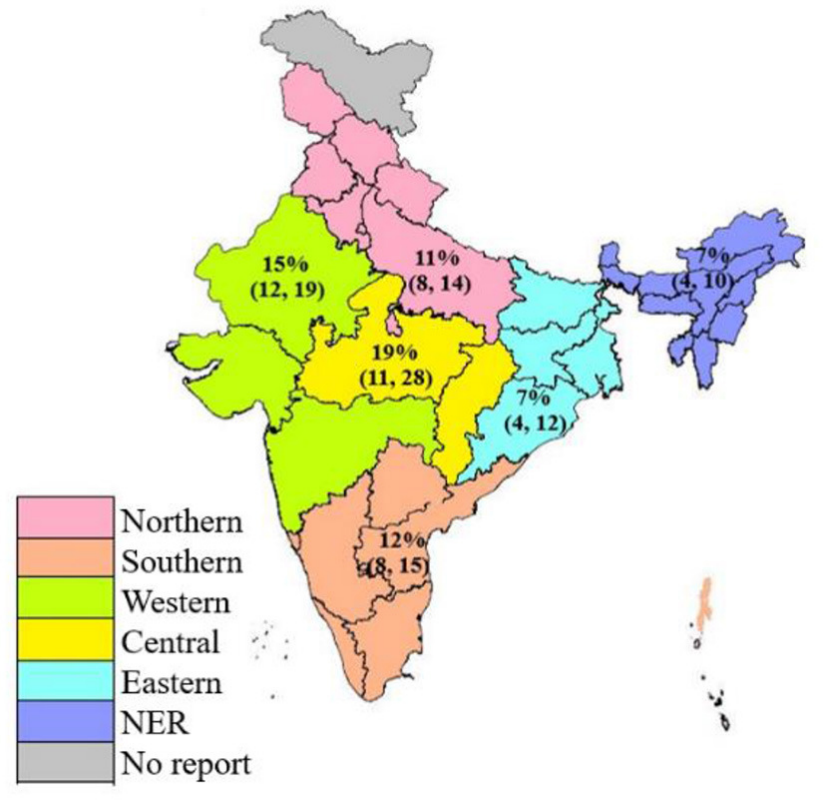
Map showing the pooled prevalence of Brucellosis in different regions of India.

Using the Random Effects model, the pooled prevalence of brucellosis in livestock of India was estimated to be 12% (95% CI: 10%−12%, *I*^2^=97%, τ^2^ = 0.0265, *p* < 0.001) from the overall 68,978 animals tested (Supplementary Figure S5). In order to study the prevalence of Brucellosis in different states of the country, studies were divided into six regions; namely Northern, Southern, Eastern, Western, Central and North-eastern. The pooled prevalence of the Northern region was found to be 11% (95 % CI: 8%−14%, *I*^2^= 96%, τ^2^ = 0.0255, *p* < 0.001), while in the Southern region the pooled prevalence of Brucellosis was 12% (95% CI: 8%−15%, *I*^2^= 97%, τ^2^ = 0.0328, *p* < 0.001). Prevalence of the disease was found to be highest in the Central and Western regions with 19% (95% CI: 11%−28%, *I*^2^= 96%, τ^2^ = 0.0409, *p* < 0.001) and 15% (95% CI: 12%−19%, *I*^2^= 97%, τ^2^ = 0.0178, *p* < 0.001), respectively. Meanwhile, the lower level of prevalence of brucellosis was seen in both the Eastern region with 7% (95% CI: 4%−12%, *I*^2^= 96%, τ^2^ = 0.0160, *p* < 0.001) and the North-eastern region with 7% (95% CI: 4%−10%, *I*^2^= 93%, τ^2^ = 0.0098, *p* < 0.001). The region-wise prevalence of Brucellosis in India is shown in Figure 3.

## Discussion

Small ruminants are socioeconomically important livestock species, ubiquitously reared as primary source of animal food and more than five million households in the country are engaged in rearing of small ruminants (15, 38). Brucellosis is a zoonotic disease that can actuate extensive human agony and vast economic losses in livestock (39). The effect of the disease on animals has significant public health implications that in turn affect feeding habits, awareness among caretakers, a consequence of lifestyles, and poor hygienic conditions leading to herd infections (40). This disease leads to low productivity, resulting in low milk production in farm animals, low herd fertility, increased abortions, and prolonged calving, thus affecting the socio-economic development of livestock owners and other animal caretakers, who are the majority in rural populations.

Systematic review and Meta-analysis help to integrate the results of a large collection of individual studies. Both are commanding tools that accumulate and sum up the information in an investigation through a statistical approach. However, it is imperative to plan and execute meta-analysis by reducing biases that may impact the outcome. The main reason for the discrepancy in meta-analysis is that it is biased on heterogeneous and often small studies (41). The subjects in the individual studies may substantially differ with respect to sample size, testing methodology, different species of livestock, geographical location, etc. (41). The literature search was performed using five main databas [PubMed, Google Scholar, Science Direct, Scopus, and Consortium for e-Resources in Agriculture (CeRA, India)] and also gray literature search by adopting abstract search and back referencing by google scholar and the language of the articles was restricted to English, and abstract being less/or no informative were rejected. Therefore, we are confident of capturing the maximum possible information for conducting the systematic review and meta-analysis. However, a meta-analysis conducted systematically may provide complementary valuable information (40). Nonetheless, by confronting such pitfalls, it is possible to arrive at a conclusion of the meta-analysis by using the trim and fill method to reduce heterogeneity among studies and arrive at a pooled prevalence value (41, 42).

The degree of heterogeneity is one of the important limitations of conducting meta-analysis. The random effect models along with subgroup analysis were the best choice during the data analysis phase to incorporate the heterogeneity. We have performed sub-group and sensitivity analysis for region-wise, diagnostic tests-wise, and species-wise variables to identify the studies contributing to heterogeneity for the further fine-tuning of prevalence rates of brucellosis. Subgroup analysis is done to investigate the heterogeneous results of particular groups, types of regions or sample sizes or types of studies (43). In the present study subgroup analysis was performed in different regions like Africa and Asian countries to determine the source of heterogeneity, heterogeneity coefficient for African countries is 0.0104 whereas for Asian countries it is 0.0149. Sensitivity analysis is a tool that tests the influence of one or more input variables on inconsistencies that can lead to anomalies in the output variable^1^. Our results of the meta-analysis depicted the prevalence estimates of brucellosis in Asia and African countries. The present study reported 8% prevalence of Brucellosis in both Asian and African countries, which is assumed to be significant for the reason that maximum livestock owners in these countries are rural marginal farmers who might not able to follow good animal husbandry practices for disease prevention and management. The high incidence of brucellosis in these countries contributed to direct and indirect economic losses and highlights the wide breadth of consequences that brucellosis has on the livelihood of livestock stakeholders in low-resource communities (39, 44). Earlier serological surveys in India showed that Brucellosis is widely prevalent in the livestock population including small ruminants throughout the regions of the country. We have unraveled the region-wise prevalence of brucellosis in India, which will aid in designing location-specific vaccination strategies to control this disease. Knowledge of prevalence and spatiotemporal distribution of the disease is of paramount importance in strict surveillance and strengthening the disease control program. This information is decisive in prioritizing the geographical regions for vaccination and implementation of other control strategies (38).

Confirmative diagnosis of brucellosis requires isolation of the causal agent which is highly hazardous and failure to isolate the pathogen is a frequent occurrence (38). Generally, MRT and RBPT are used for brucellosis screening, especially in developing countries where other tests are crucial to organize on a large scale, as special equipment and training are needed. Other confirmatory tests such as ELISA, CFT, and SAT in combination with MRT and RBPT are conducted. ELISA is an available assay for use on milk and serum and is very useful where a large number of samples require testing, sometimes milk ELISA is used on pooled samples, which is more cost-effective than testing individual animals (40, 45). In our study, diagnostic tests like PCR, CFT, and RBPT have detected more positive cases on average indicating that a combination of serological tests or serial test procedures should be adopted to reduce the number of both false-negative and false positives.

Higher disease prevalence in cattle in comparison to buffaloes has been reported by other researchers (44). This is in accordance with our study showing that the prevalence of brucellosis was highest in cattle followed by sheep which might be due to free grazing and movement of herds and flocks which contribute to the wide distribution of brucellosis in these animals and to other animal species (46, 47). Other reports have shown that animal movement and grazing in common pastures have a significant correlation with the seroprevalence with *p* < 0.001 (41, 48). Some risk factors like shared communal pastures for grazing and animal movement were cited for Brucellosis sero-positivity (42).

Estimating the prevalence of brucellosis is important step in designing nationwide veterinary health response to this pathogen, these estimations suggest that well-planned surveillance studies in different geographic settings are required to generate reliable data on disease burden including an economic loss in Asian and African countries in spatiotemporal pattern, so as to create a proper distribution of research and prevention effort.

## Conclusions

The prevalence of Brucellosis in Asian and African countries has been estimated based on systemic review and meta-analysis. Efforts were made using statistical methods to avoid biases and heterogeneity in estimating prevalence value etc. It was possible to identify the research gaps in understanding Brucellosis epidemiology thereby suggesting the need to perform robust surveillance programs and control measures to combat the disease. These programs would further enable us to estimate the species-specific prevalence rate of Brucellosis in endemicity-prone countries that would serve as an alternative to primary and secondary infections using mathematical models. Our findings suggest that well-planned surveillance studies in different geographic settings are also needed to generate reliable data on disease burden including economic loss in Asian and African countries. Efforts are made in this study to avoid bias by including research, using an adequate statistical methodology, and interpreting the results based on the context and available evidence. Meta-analysis represents a powerful way to summarize and effectively increased the sample size to provide a more valid pooled estimate.

## Data availability statement

The original contributions presented in the study are included in the article/Supplementary material, further inquiries can be directed to the corresponding author.

## Author contributions

KS carried out the literature search, analyzed the data, carried out the meta-analysis in R software, interpreted the data, and wrote the draft of manuscript. SP and RA designed and conceptualized the idea. HD and AN rewritten the draft and edited the manuscript. SR, CS, and AC provided guidance and support to carry out the research. All authors contributed to the article and approved the submitted version.

## Conflict of interest

Author CC was employed by Midwest Veterinary Services, Inc. The remaining authors declare that the research was conducted in the absence of any commercial or financial relationships that could be construed as a potential conflict of interest.

## Publisher's note

All claims expressed in this article are solely those of the authors and do not necessarily represent those of their affiliated organizations, or those of the publisher, the editors and the reviewers. Any product that may be evaluated in this article, or claim that may be made by its manufacturer, is not guaranteed or endorsed by the publisher.

## Footnotes

^1^Available online at: https://www.javatpoint.com/list-of-r-packages (accessed December 23, 2020).

^2^Available online at: https://www.statsdirect.com/help/meta_analysis/heterogeneity.htm (accessed on 7 January 2021).

## Supplementary material

Supplementary File S1 PRISMA checklist.

Supplementary Figure S2 Forest plot. Continent wise analysis.

Supplementary Figure S3 Forest plot. Diagnostic test -wise analysis.

Supplementary Figure S4 Forest plot. Species-wise analysis.

Supplementary Figure S5 Forest plot. Prevalence of brucellosis in India.

Supplementary File S6 Quality assessment of studies by Authors. Average score of two independent authors and Aiken agreement Index.
