# Supplementary material for: Prevalence of brucellosis in livestock of African and Asian continents: A systematic review and meta-analysis
Source: Front Vet Sci. 2022 Sep 9;9:923657. doi: 10.3389/fvets.2022.923657 (PMC9500530; doi:10.3389/fvets.2022.923657)
Supplement: Supplementary File S1 — PRISMA checklist. [file Data_Sheet_1.docx]

1. Abbas BA, Aldeewan AB. Occurrence and epidemiology of Brucella spp. in raw milk samples at Basrah province, Iraq. Bulgarian J Vet Med. 2009;12:136–142.
2. Adamu SG, Atsanda NN, Tijjani AO, Usur AM, Sule AG, Gulani IA. Epidemiological study of bovine brucellosis in three senatorial zones of Bauchi State, Nigeria. Vet World. 2016;9(1):48–52.
3. Adamu S, Tijjani A, Atsanda N, Adamu N. Serological survey of Brucella antibodies in cattle breeding herds in northeastern Nigeria. J Vet Adv. 2014;4(7):599.
4. Ahmad T, Khan I, Razzaq S, Khan S-U-H, Akhtar R. Prevalence of bovine brucellosis in Islamabad and Rawalpindi districts of Pakistan. Pak J Zool. 2017;49(3):1123–6.
5. Aiyedun J, Oludairo O, Olorunshola I, Furo N, Olowoleni F, Adam M, et al. Seroepidemiological survey of bovine brucellosis in selected Fulani Herds in Kwara State, Nigeria. J Adv Vet Anim Res. 2017;4(2):1.
6. Akhtar R, Chaudhry ZI, Shakoori AR, Ahmad MU, Aslam A. Comparative efficacy of conventional diagnostic methods and evaluation of polymerase chain reaction for the diagnosis of bovine brucellosis. Vet World. 2010;3:53–56.
7. Alhaji NB, Wungak Y. Epizootiological survey of bovine brucellosis in nomadic pastoral camps in Niger state, Nigeria. Nigerian Vet J. 2013;18:34.
8. Al-Mariri A. Isolation of Brucella melitensis strains from Syrian bovine milk samples. Bulg J Vet Med. 2015;18(1):40–8.
9. Angara TEE, Ismail AA. Sero-prevalence of bovine brucellosis in Kuku Dairy Scheme, Khartoum North, Sudan [Internet]. Psu.edu. [cited 2021 Oct 13]. Available from: http://citeseerx.ist.psu.edu/viewdoc/download?doi=10.1.1.547.9531&rep=rep1&type=pdf
10. Aulakh HK, Patil PK, Sharma S, Kumar H, Mahajan V, Sandhu KS. A study on the epidemiology of bovine brucellosis in Punjab (India) using milk-ELISA. Acta Vet Brno. 2008;77(3):393–9.
11. Belal S, Ansari A. Seroprevalence of *Brucella abortus* antibodies in the cattle population in the selected upazilas of Sirajgonj district. Banglad J Vet Med. 2014;11(2):127–30.
12. Bertu WJ, Ducrotoy MJ, Muñoz PM, Mick V, Zúñiga-Ripa A, Bryssinckx W, et al. Phenotypic and genotypic characterization of Brucella strains isolated from autochthonous livestock reveals the dominance of B. abortus biovar 3a in Nigeria. Vet Microbiol. 2015;180(1–2):103–8.
13. Brahmabhatt MN, Varasada RN, Bhong CD, Nayak JB. Seroprevalence of Brucella spp. in buffaloes in the central Gujarat region of India. Buffalo Bull. 2009;28:73–75.
14. Bakhtullah, Basit A, Shahid M, Parveen F, Gul S, Khan MA, Wazir I, Raqeebullah, Rahim K (2014). Sero–
15. prevalence of brucellosis in cattle in southern area of Khyber Pakhtunkhwa, Pakistan. Res. J. Vet. Pract. 2 (4): 63 – 66
16. Cadmus SIB, Adesokan HK, Adedokun BO, Stack JA. Seroprevalence of bovine brucellosis in trade cattle slaughtered in Ibadan, Nigeria, from 2004-2006. J S Afr Vet Assoc. 2010;81(1):50–3.
17. Cadmus SIB, Adesokan HK, Stack J. The use of the milk ring test and rose bengal test in brucellosis control and eradication in Nigeria. J S Afr Vet Assoc. 2008;79(3):113–5.
18. Cadmus SIB, Alabi PI, Adesokan HK, Dale EJ, Stack JA. Serological investigation of bovine brucellosis in three cattle production systems in Yewa Division, south-western Nigeria. J S Afr Vet Assoc. 2013;84(1):E1-6.
19. Cadmus SIB, Ijagbone IF, Oputa HE, Adesokan HK, Stack JA. Serological survey of Brucellosis in livestock animals and workers in Ibadan, Nigeria. Afr J Biomed Res [Internet]. 2009;9(3). Available from: <http://dx.doi.org/10.4314/ajbr.v9i3.48900>
20. Chand P, Sharma AK. Situation of brucellosis in bovines at organized cattle farms belonging to three different states. J Immunol Immunopathol. 2004;6:11–15.
21. Deepthi B, Ramani Pushpa RN, Srivani M, Kavitha KL, Subramanyam KV. Seroprevalence patterns of bovine brucellosis in organised and unorganised farms of coastal Andhra pradesh, India. ijah. 2018;57(2):175.
22. Degefu H, Mohamud M, Hailemelekot M, Yohannes M. Seroprevalence of bovine brucellosis in agro pastoral areas of Jijjiga zone of Somali National Regional State, Eastern Ethiopia. Ethiop Vet J [Internet]. 2011;15(1). Available from: <http://dx.doi.org/10.4314/evj.v15i1.67683>
23. Dey SK, Rahman MS, Rima UK, Hossain MZ, Chowdhury GA, Pervin M, et al. Serological and pathological investigation of brucellosis in dairy cows of Mymensingh district, Bangladesh. Banglad J Vet Med. 2014;11(2):107–12.
24. El-Gohary A, Mohamed A, Al-Sherida Y. Seroprevalence of brucellosis and typing of Brucella melitensis biovar 2 in lactating cows in Kuwait. J Adv Vet Ani Res. 2016;3:229–235.
25. Etman RH, Barsoum SA, Ibrahim IGA, El-Ashmawy WR, Abou-Gazia KA. Evaluation of efficacy of some serological tests used for diagnosis of brucellosis in cattle in Egypt using latent class analysis. Sokoto J Vet Sci. 2014;12(2):1–7.
26. Genç O, Otlu S, Şahin M, Aydin F, Gökce HI. Seroprevalence of brucellosis and leptospirosis in aborted dairy cows. Turkish J Vet Ani Sci. 2005;29:359–366.
27. Ghodasara SN, Roy A, Bhanderi BB. Comparison of Rose Bengal plate agglutination, standard tube agglutination and indirect ELISA tests for detection of Brucella antibodies in cows and buffaloes. Vet World. 2010;3:61.
28. Gogoi SB, Hussain P, Sarma PC, Barua AG, Mahato G, Bora DP, et al. Prevalence of bovine brucellosis in Assam, India. J Entomol Zool Stud. 2017;5:179–185.
29. Gul ST, Khan A, Rizvi F, Hussain I. Sero-prevalence of brucellosis in food animals in the Punjab, Pakistan. Pak Vet J. 2014;34:454–458.
30. Minda AG, Gobena A, Tesfu K, Getachew T, Angella A, Gezahegne MK. Seropositivity and risk factors for Brucella in dairy cows in Asella and Bishoftu towns, Oromia Regional State, Ethiopia. Afr J Microbiol Res. 2016;10(7):203–13
31. Hassan AA, Uddin MB, Islam MR, Cho H-S, Hossain MM. Serological prevalence of brucellosis of cattle in selected dairy farms in Bangladesh. Taehan Suui Hakhoe Chi Taehan Suui Hakhoe. 2014;54(4):239–43.
32. Islam MS, Islam MA, Khatun MM, Saha S, Basir MS, Hasan M-M. Molecular detection of Brucella spp. From milk of seronegative cows from some selected area in Bangladesh. J Pathog. 2018;2018:1–7.
33. Jackson R, Ward D, Kennard R, Amirbekov M, Stack J, Amanfu W, et al. Survey of the seroprevalence of brucellosis in ruminants in Tajikistan. Vet Rec. 2007;161(14):476–82.
34. Jain-Gupta N, Waldrop SG, Tenpenny NM, Witonsky SG, Boyle SM, Sriranganathan N. Rough Brucella neotomae provides protection against Brucella suis challenge in mice. Vet Microbiol. 2019;239(108447):108447.
35. Jamil T, Melzer F, Saqib M, Shahzad A, Khan Kasi K, Hammad Hussain M, et al. Serological and molecular detection of bovine brucellosis at institutional livestock farms in Punjab, Pakistan. Int J Environ Res Public Health. 2020;17(4):1412.
36. Kala MS, Sankhala LN, Champawat M, Puniya SR, Shah NM. Comparative efficacy study of different serological tests for the diagnosis of brucellosis in bovines. Pharma Innov. 2018;7:562.
37. Kaushik P, Kumar M, Anjay K, S. K, P. Sero-prevalence of Bovine Brucellosis in Bihar, India. J Vet Pub Hlth. 2016;14:51–53.
38. Kebede T, Ejeta G, Ameni G. Seroprevalence of bovine brucellosis in smallholder farms in central Ethiopia (Wuchale-Jida district. Revue de Médecine Vétérinaire. 2008;159:3.
39. Khan A, Gul ST, Saleemi MK, Du X. Seroprevalence of brucellosis in cattle (Bos Taurus) kept in Peri urban areas of Pakistan. AAgrobiol Records. 2020;1:6–10.
40. Kolo FB, Adesiyun AA, Fasina FO, Katsande CT, Dogonyaro BB, Potts A, et al. Seroprevalence and characterization of Brucella species in cattle slaughtered at Gauteng abattoirs, South Africa. Vet Med Sci. 2019;5(4):545–55.
41. Krishnamoorthy P, Patil SS, Shome RA, Rahman H. Seroepidemiology of Infectious bovine rhinotracheitis and brucellosis in organized dairy farms in southern India. Indian J. Ani. Sci. 2015, 85, 695–700.
42. Kumar VN, Bharathi MV, Porteen K, Sekar M. Serological studies on bovine brucellosis of Tamil Nadu. Indian Vet J. 2017;94:68–70.
43. Lobna MA, Khoudair SM, Osman SA. Sero-diagnosis of brucellosis by using simple and rapid field tests with emphasis on some possible risk factors in humans. Glob Vet. 2014;12:320–325.
44. Mehra KN, Dhanesar NS, Chaturvedi VK. Sero-prevalence of brucellosis in bovines of Madhya Pradesh. Indian Vet J. 2000;77:571–573.
45. Mittal V, Kumar M, Ambwani T. Seroepidemiological pattern of brucellosis among livestock of district Udham Singh Nagar in Uttaranchal. Indian J Vet Med. 2005;25:28.
46. Mohamud Aİ, Mohamed YA, Mohamed MI. Sero-prevalence and associated risk factors of bovine brucellosis in selected districts of Benadir Region, Somalia. J istanb vet sci. 2020;57–63.
47. MohamedKhair W, Elfadil A, Elgadal A, Shuaib Y. Seroprevalence and risk factors of anti-brucella antibodies in cattle in Khartoum State, the Sudan. J Adv Vet Anim Res. 2016;3(2):134.
48. Negash W, Reath G, Terefe G, Mamo G. Seroprevalence of bovine brucellosis and its associated risk factors in Jikow District. Vol. 4. Ethiopia: Gambella Regional State; 2020.
49. Nitu M, K. S, Mohan K. Sero-epidemiological and therapeutic aspects of brucellosis (Brucella abortus) in Cattle & Buffaloes. J Ani Res. 2013;3:65–74.
50. Otlu S, Sahin M, Atabay HI, Unver A. Serological investigations of brucellosis in cattle, farmers and veterinarians in the kars district of turkey. Acta Vet Brno. 2008;77(1):117–21.
51. Pandeya YR, Joshi DD, Dhakal S, Ghimire L, Mahato BR, Chaulagain S, et al. Seroprevalence of brucellosis in different animal species of Kailali district, Nepal. Int J Infect Microbiol. 2013;2(1):22–5.
52. Pathak AD, Dubal ZB, Karunakaran M, Doijad SP, Raorane AV, Dhuri RB, et al. Apparent seroprevalence, isolation and identification of risk factors for brucellosis among dairy cattle in Goa, India. Comp Immunol Microbiol Infect Dis. 2016;47:1–6.
53. Priyadarshini A, Sarangi LN, Palai TK, Panda HK, Mishra R, Behera PC. Brucellosis in cattle and occupationally exposed human beings: A serosurvey in Odisha. India;
54. Md. Siddiqur Rahman. Brucellosis among ruminants in some districts of Bangladesh using four conventional serological assays. Afr J Microbiol Res [Internet]. 2012;6(22). Available from: http://dx.doi.org/10.5897/ajmr12.475
55. Reddy RR, Prejit SB, Vinod VK, Asha K. Seroprevalence of brucellosis in slaughter cattle of Kerala, India. J Foodborne Zoo Dis. 2014;2:27–29.
56. Saeed U, Ali S, Khan TM, El-Adawy H, Melzer F, Khan AU, et al. Seroepidemiology and the molecular detection of animal brucellosis in Punjab, Pakistan. Microorganisms. 2019;7(10):449.
57. Salem L, Khalifa N, Khoudair, Moustafa S. Sero-diagnosis of brucellosis in Gharbiya governorate, Egypt. Benha Veterinary Medical Journal. 2016;31(1):10–6.
58. Samaha H, Al-Rowaily M, Khoudair RM, Ashour HM. Multicenter study of brucellosis in Egypt. Emerg Infect Dis. 2008;14(12):1916–8.
59. Samaha H, Mohamed TR, Khoudair RM, Ashour HM. Serodiagnosis of brucellosis in cattle and humans in Egypt. Immunobiology. 2009;214(3):223–6.
60. Sarker MAS, Begum MM, Rahman MF, Islam MT, Yasmin L, Ehsan MA, et al. conventional pcr based detection of brucella abortus infected cattle in some selected areas of bangladesh. Banglad J Vet Med. 2018;16(1):39–44.
61. Sarker MA, Rahman MS, Begum MM, Rahman MB, Rahman MF, Neubauer H, et al. Milk ring, rose bengal tests and conventional PCR based detection of Brucella abortus infected dairy cattle in Bangladesh. African J Microbiol Res. 2017;11:1505–1509.
62. Sarker MAS, Rahman MS, Islam MT, Rahman A, Rahman MB, Rahman MF. Prevalence of brucellosis in dairy cattle in organized and smallholder farms in some selected areas of Bangladesh. Banglad J Vet Med. 2014;12(2):167–71.
63. Selim A, Attia K, Ramadan E, Hafez YM, Salman A. Seroprevalence and molecular characterization of Brucella species in naturally infected cattle and sheep. Prev Vet Med. 2019;171(104756):104756.
64. Abdalla Senein M. Serological survey of cattle brucellosis in Eldein, eastern Darfur, Sudan. Afr J Microbiol Res [Internet]. 2012;6(32). Available from: <http://dx.doi.org/10.5897/ajmr12.653>.
65. Senthil NR, Narayanan SA. Seroprevalence study of bovine brucellosis in slaughter house.
66. Shabbir MZ, Nazir MM, Maqbool A, Lateef M, Shabbir MAB, Ahmad A, et al. Seroprevalence of Neospora caninum and Brucella abortus in dairy cattle herds with high abortion rates. J Parasitol. 2011;97(4):740–2.
67. Subedi S, Prajapati M, Dhakal B. Sero-surveillance of Brucellosis in cattle of Chitwan district, Nepal. Int J Appl Sci Biotechnol. 2016;4(3):365–71.
68. Sunder J, Rai RB, Kundu A, Chatterjee RN, Senani S, Kumar SJ. Incidence and prevalence of livestock diseases of Andaman and Nicobar Islands. Indian J Ani Sci. 2005;75:1041.
69. Tesfaye G, Wondimu A, Asebe G, Regasa F, Mamo G. Sero-prevalence of bovine brucellosis in and around kombolcha, Amhara regional state, Ethiopia. Mycobact Dis [Internet]. 2017;07(02). Available from: http://dx.doi.org/10.4172/2161-1068.1000242
70. Verma S, C. KR, M. S, Nigam P. Abortions and infertility in livestock due to brucellosis in Himachal Pradesh, India. Vet Arhiv. 2000;75–82.
71. Vhoko K, Iannetti S, Burumu J, Ippoliti C, Bhebhe B, De Massis F. Estimating the prevalence of Brucellosis in cattle in Zimbabwe from samples submitted to the Central Veterinary Laboratory between 2010 and 2014. Vet Ital. 2018;54(1):21–7.
72. Yuguda MU, Sundaram S, Lakshmanan G, Ayyasamy E, Purushothaman S, Kannan P, et al. Serosurveillance of bovine brucellosis using RBPT and c-ELISA and comparative evaluation of test performance. Int J Curr Microbiol Appl Sci. 2019;8(08):559–68.
73. Zubairu A, Ardo MB, Mai HM. Seroprevalence of ruminant brucellosis in three selected local government areas of Taraba state. Sokoto J Vet Sci. 2014;12(1):51.
