# Supplementary figures and images for: Prevalence of brucellosis in livestock of African and Asian continents: A systematic review and meta-analysis
Source: Front Vet Sci. 2022 Sep 9;9:923657. doi: 10.3389/fvets.2022.923657 (PMC9500530; doi:10.3389/fvets.2022.923657)

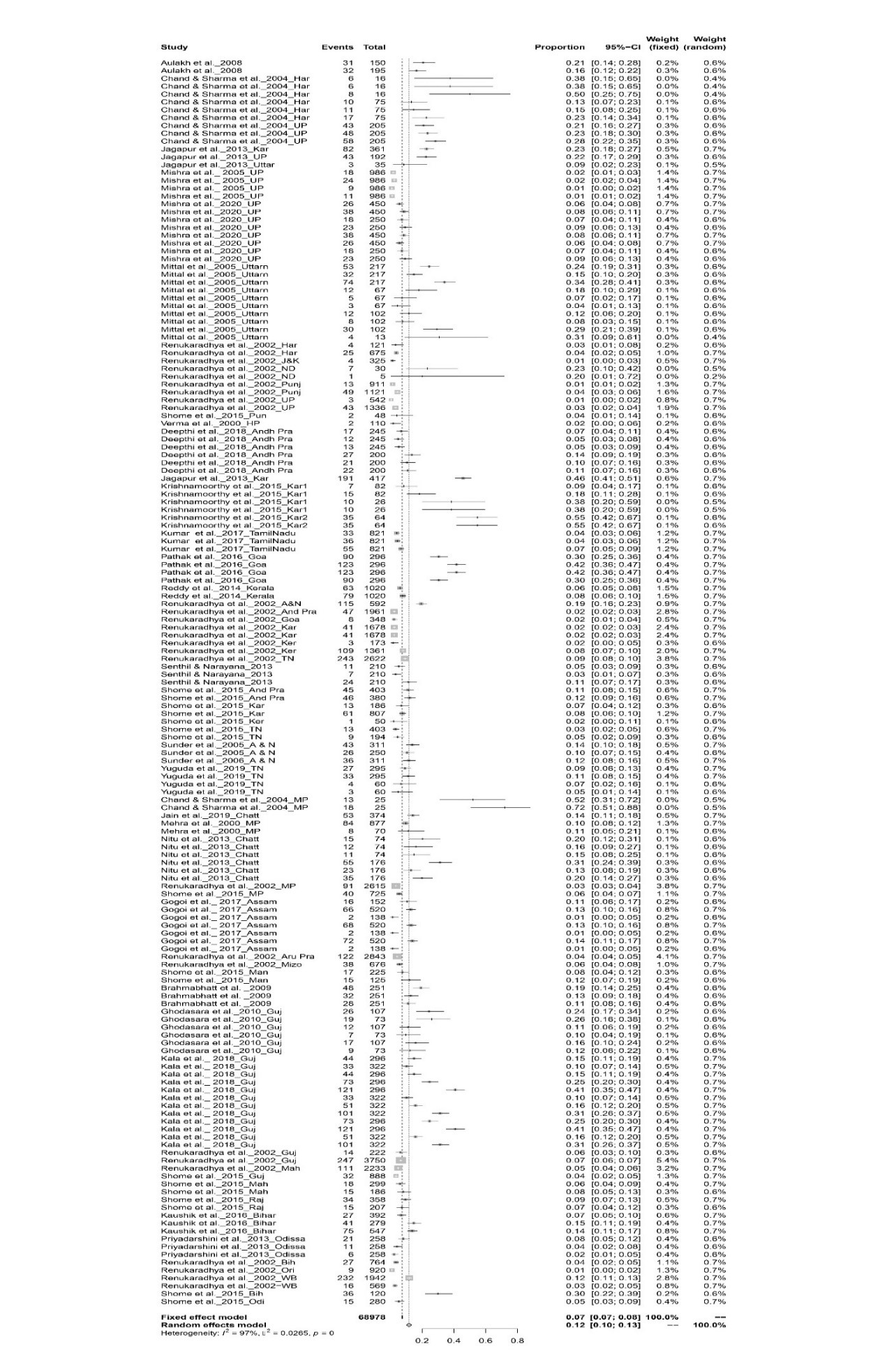

Supplement: Supplementary Figure S2 — Forest plot. Continent wise analysis. [file Data_Sheet_1.ZIP › Supplementary files +18 APR 2022/Supp.Fig__S5.tiff]
